# Assessment of Factors Influencing the Implementation of Biosecurity Measures on Pig Farms in the Western Highlands of Cameroon (Central Africa)

**DOI:** 10.1155/2018/9173646

**Published:** 2018-05-27

**Authors:** Marc K. Kouam, Junior O. Moussala

**Affiliations:** ^1^Department of Animal Production, Faculty of Agronomy and Agricultural Sciences, PO BOX 188, Dschang, Cameroon; ^2^Center for Research on Filariasis and Other Tropical Diseases (CRFilMT), P.O. BOX 5797, Yaoundé, Cameroon

## Abstract

Biosecurity plays an irreplaceable role in preventing diseases and increasing productivity on farm. The main objective of this study was to characterize pig farming and investigate factors influencing biosecurity on pig farms in the western highlands of Cameroon. Data were collected from May to July 2017 using a questionnaire and observations. A technical scoring system was developed from the biosecurity measures. The results revealed that most farmers are males (76.29%), on average 47.82 ± 10.34 years old, with secondary school level (53.61%). The most common husbandry system is extensive (73.22%). Over a total score of 93, measures with higher scores (>80) included “employees do not rear pigs at home,” “animals of different age not in the same room,” “unsold animals from market quarantined prior to reintroduction into the herd,” “production materials not exchanged among farms,” “piggeries clean every day,” “disinfectants used,” “pigs vaccinated,” and “vaccination calendar respected.” Those with the lowest score (<6) were “sanitary lock present,” “use of herd specific clean coveralls and boots on farm,” and “entry restriction sign post present.” The biosecurity level was associated with production system, with the score 6.57 and 3.66 points lower for extensive and semi-intensive farms, respectively, than for intensive system. Farmer's age, gender, education level, and herd size did not affect the level of biosecurity. The results can be used to improve the general biosecurity status in pig herds in the country which in turn will lead, as observed elsewhere, to improved technical performance and economic gain.

## 1. Introduction

Cameroon's population is fast growing, as documented in a previous report [1] indicating a shift of a population from 12.1 million inhabitants in 1990 to 22.8 million inhabitants in 2014. With this growing population in the country, the demand for meat is very high. For instance, in 2009, pig farming in Cameroon provided annually only 30,000 tons of meat while the prevision was estimated at 42,000 tons for that year [2]. That year, pork consumption was estimated at 1.8 kg per inhabitant per year (kg/inh/year) and projected to be 2.0 kg/inh/year in 2015 and 2.5 kg/inh/year in 2025 [2]. Of the 265,816 tons of meat produced in 2013, pork contribution was 35180 tons only [3]. Among the constraints limiting pig productivity, diseases have the lion's share [2, 4]. Some of the diseases reported to occur include African swine fever (ASF), classical swine fever, Aujeszky's disease, enteritis, transmissible gastroenteritis, porcine encephalomyelitis, erysipelas, dysentery, pasteurellosis, tuberculosis, salmonellosis, and parasitic diseases [2, 5]. For example, because of ASF, pig productivity dropped from 41,043 to 35,180 tons of meat in 2012 and 2013, respectively [3].

Different means have been developed to control these diseases in pigs and other food animals including biosecurity, vaccination, surveillance, and culling of the animals. Biosecurity, defined as a set of management practices or measures to prevent introduction and spread of pathogens within and between farms [6, 7], has been reported to be the cheapest way to control diseases in flocks or herds. However, many factors have been reported to affect the adoption and correct implementation of biosecurity on farm. Ajewole and Akinwumi [8] reported that the level of education, farm size, and training in animal production all have significant positive influence on the poultry farms' biosecurity control score while age, number of household labor, and distance from the nearest poultry farm show significant negative influence on the farms' biosecurity control score. Similarly, Can and Altuğ [9] found that herd size and producers' education level were positively correlated with biosecurity score on dairy cattle farms. Previous studies on pig herds provided data on biosecurity practices only [10, 11] while some also addressed the question of factors influencing the implementation of biosecurity measures. Postma et al. [12] found that biosecurity status in pig farms in four European countries was significantly associated with the number of pathogens vaccinated against, with more weaned piglets per sow per year, and with the estimated frequency of treatment against certain clinical signs of disease. Sociotechnical factors such as “the female caretaker in the farrowing unit,” “a farmer with fewer years of experience,” “more educated personnel,” “age of buildings,” and “herd size” have been found to be associated with the biosecurity status in pig herds [11, 13].

To our knowledge, such factors have not yet been investigated in pig farming in Cameroon. Therefore, the objectives of this study were to describe the characteristic of pig farming, to determine the biosecurity score, and to investigate the socioeconomic and technical characteristics of farms and farmers affecting the implementation of biosecurity measures in the western highlands of Cameroon.

## 2. Materials and Methods

### 2.1. Study Area

**Figure 1:**
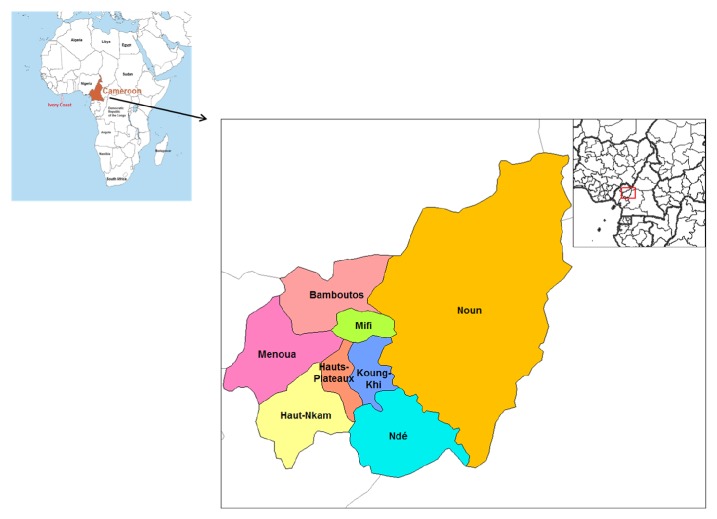
Map of the West Region of Cameroon showing Menoua Division.

The study was carried out from May to July 2017 on pig farms located in Menoua Division (Figure 1) in the West Region of Cameroon. The area lies between longitude 9°49′–10°20′ east of the Greenwich meridian and latitude 5°17′–6°22′ north of the equator. The region is characterized by a typical climate with two main seasons, the dry season ranging from November to mid-March and the rainy season which prevails from mid-March to October. Temperature ranges between 15° and 24°C [14]. Livestock species include pigs, small ruminants (sheep, goat), cattle, domestic cavies* (Cavia porcellus)*, and poultry. The West Region is one of the highest pig production regions of the country, and one of the foci of ASF outbreak in the country [2, 4]. The last outbreak of ASF dates back to 2016, and the most recent report on pig population in the West Region gives an estimated pig population of 155,000 heads in 2016; these data were obtained from the regional authorities of the Ministry of Livestock, Fisheries and Animal Industry (MINEPIA) of the West Region.

### 2.2. Questionnaire Design

The questionnaire was divided into three sections. The first question set consisted of socioeconomic characteristic of farmers (age, sex, education, farming, and experience, among others). The second part was related to production characteristics such as farm size, breed, and production system. The third section was made up of questions dealing with biosecurity components including isolation, traffic control, and sanitation, as defined by FAO/OIE/World Bank [15]. Before starting the field work, the questionnaire was pretested and the questions were adjusted accordingly. Pretesting of the questionnaire was carried out by the investigators among a small sample of pig farmers. Adjustments were made by replacing some words, deleting irrelevant questions, and reformulating and splitting some questions.

### 2.3. Selection of Farms

The areas to sample were chosen under the guidance of the veterinary health officials as the subdivisions where pig husbandry was known to take place. Given that previous reliable data on pig farmers were not available, all pig farms within the sample area were included in the study. In the absence of official registry of pig farms, farms were first located with the help of the local veterinary health officials. The next farms were located using the snow ball technique (the manager of the previously located farm helped to identify the next farm and so on until the whole area was covered). According to the regional authorities of the MINEPIA of the West Region, Menoua Division is one of the highest pig farming areas of the West Region. The questionnaire survey was carried out through a face-to-face interview between the researchers and the pig farm manager and through personal observations of the researchers. Only the pig farm manager was eligible for interview.

### 2.4. Biosecurity Scoring System

A technical scoring system was developed from the biosecurity indicators (measures), ranging from 0 to 1. A biosecurity measure was coded as 1 if this measure is present (implemented), or 0 if the measure is absent (not implemented) [9, 16, 17]. To obtain the final score for each measure, all the values recorded on farms (either 0 or 1 per farm) were added up. The measures were grouped into sections, each section corresponding to a biosecurity component (isolation, traffic control, and sanitation). Since a component is made up of several measures, the scores of individual measures were added up to generate the mean score for the component, by dividing the total score by the total number of measures within a component. The maximum score for a given measure (biosecurity indicator) was 97 points matching the total number of farms under investigation while the maximum score for a given farm was 31 matching the total number of measures investigated.

### 2.5. Statistical Analysis

The farmer's related characteristics were examined using descriptive statistics (frequency and mean). The ANOVA test was used to test the effect of biosecurity component on biosecurity measures score. The test aimed at assessing whether the implementation level of biosecurity was the same among the three components of biosecurity (isolation, traffic control, and sanitation). The multivariate linear regression model was used to evaluate the relation between the biosecurity score of farms and the socioeconomic and technical characteristics (factors) of farmers and farms. To perform the analysis, qualitative variables were coded by creating dummy variables with two values, either 1 or 0, while considering the reference level for the factor as the level with the lowest value. Collinearity was checked by looking at the values of the “Tolerance” and the Variance Inflation Factor (VIF) of variables; a “Tolerance” < 0.1 is indicative of redundancy of a variable, and a VIF greater than 10 indicates a collinearity problem. Data were analyzed using the SPSS statistical package (version 13.0, SPSS Inc., USA), and the significance level was fixed as 5%.

## 3. Results

### 3.1. General Characteristics of Farmers and Farms

The general characteristics of pig farmers and farms are presented in Table 1. This table showed that most farmers are males (76.29%), crop producers, on average 47.82 ± 10.34 years old (range: 32–67), married (65.96%), have schooled up to secondary level (53.61%), and have been trained in animal husbandry (70.10%); animal husbandry referred to animal rearing in general, irrespective of the species. The highest proportion of farmers is Christian (79.46%) and the lowest Muslim (0.86%). The average number of years farmers had experience in rearing animals was 10.86 ± 6.42 years (range: 1–22). The most common husbandry system was extensive (73.22%) while the mean herd size was 16.87 ± 11.04 pigs (range: 2–41) and the mean farm age 8.18 ± 5.56 years (range: 2–22); farm age referred to years since start of the farm, not age of buildings.

### 3.2. Score of Biosecurity Indicators and Components

The score of indicators of biosecurity, as well as the mean score for each biosecurity component, is shown in Table 2. For indicators belonging to the isolation component of biosecurity, those with high score (>50) included distance between two farms greater than 500 m, quarantine of new animals, keeping animals of the same age in the same rooms, and absence of pigs at employees' home. The indicator with the poorest score was the use of herd specific clean coverall and boots by employees on farm (6 points over 97). The score for indicators pertaining to traffic control varied from 0 to 93 points for presence of entry restriction sign post and nonexchange of production materials (feeders, drinkers, shovel, wheelbarrow, and broom, among others) among farms, respectively. Measures with high score (>50) also included assignment of each employee to a specific building, quarantine of unsold pigs returning from a market, and use of boars from own farm; quarantine period usually took a few days (2 days onwards) during which animals, kept in a separate stable, were observed for any change in behavior and any sign of disease. Indicators under sanitation had scores ranging from 0 to 90. The score of half of the indicators was below 50. Of these measures, two had scores lower than 5, namely, presence of sanitary lock (score = 0/97) and disinfection of vehicles entering the farm (score = 4/97). For the remaining half, two indicators had score greater than or equal to 90, including the daily cleaning (floor, drinkers and feeders cleaned from dirt, and dung and feed waste each morning before feed supply) of the piggery and use of disinfectants. The ANOVA test of the effect of biosecurity component on the score of biosecurity indicators revealed that this effect was not significant (*p* = 0.89); indeed the mean scores of biosecurity components were not significantly different.

### 3.3. Factors Influencing the Use of Biosecurity Measures

The results of multivariate regression analysis of factors affecting the implementation of biosecurity measures are presented in Table 3. From this table, negative significant relationship was established between production systems and farm biosecurity score. The biosecurity score was 6.57 and 3.66 points lower for extensive and semi-intensive farms, respectively, than for intensive system.

## 4. Discussion

This study is the first one describing the characteristics of pig farmers and pig farms, as well as the implementation of certain biosecurity measures on pig farms and factors influencing the implementation of biosecurity measures on the farm within the study area. In order to collect reliable data, face-to-face interviews and field observations were used to complete the questionnaire rather than using mailed questionnaires.

The results of this work indicated that the main actors in pig farming are men above forty years old, which disagree with the findings on Swedish pig farms [11] where the proportion between men and women pig farms managers is balanced. Concerning the age of pig farm manager, our findings agree with those on Swedish pig farms [11] where the mean age should be around 40, based on the median of 23 years (range: 5–41 years) of experience in pig farming. The old age of persons in charge of pigs in Cameroon might be related to the high schooling rate of the population in the study area where young people mostly spend their time at school and thus cannot properly take care of animals. In the country also, animal husbandry is mostly carried out by men rather than women, who are mostly involved in crop production. Men's deep involvement in animal husbandry rather than in crop farming is cultural and is inherited from tradition. The majority of people responsible for pigs had secondary school education level, with 10 years of experience in animal husbandry, but were not formally trained in animal husbandry. In comparison with the Swedish pig farmers [11], Cameroon pig farmers also had the relevant years of experience and the education level but not the educational background (training in animal husbandry) required in pig husbandry. That most pig farmers in Cameroon were not trained in animal production is a flaw for pig industry in the country. Animal husbandry was not the main activity of pig farmers within the study area but crop production. Other main occupations also included trade or office work (civil servants); a minority were retired. This picture gives the impression that pig farming alone does not allow the farmers to make a living within the study area. The reason for this may be related to recurrent epidemics of ASF that has been decimating the herds in different localities of the country. Concerning farmers' religion, the lowest proportion of farmers were Muslims probably due to religious restrictions. Though Muslims are known to avoid pork and are often overlooked when addressing questions dealing with pig industry, this study showed that they should be reckoned with not as consumers, but as producers. The mean herd size was 16.87 ± 11.04, with a minimum of 2 and a maximum of 41. Costard et al. [18] reported a minimum herd size of 2 and a maximum of 98 in Madagascar. In spite of the maximum herd size in Madagascar greater than the maximum size in Cameroon, the median size in Madagascar varying from 3 to 7 suggests that the herd size and the production systems in both countries are the same. This may be partly due to the common disease challenges faced by pig industry in both countries, especially the regular outbreaks of contagious diseases, such as ASF [2, 19–21]. In fact, the high mortality rate of these diseases (close to 100% for ASF) in affected farms causes farm owners either to remain only with the few survivors, to restock the farms with few animals, or to take less risk in investing for a larger farm.

The biosecurity scoring system adopted in this work was a linear scoring system (each measure is equally scored) [16, 17, 22] rather than a risk based weighted scoring system [13, 23]. Authors supporting the weighted scoring system consider that each pathway of disease transmission has a different or peculiar efficiency [13, 23]; their standpoint is supported by some examples such as the fact that direct contact between animals (contact between healthy animals and diseased animals newly purchased) poses a higher risk whereas indirect contacts (transmission of pathogens through rodents or tools sharing between farms) are less efficient in the transmission of pathogens. Though they are right, the linear scoring system was preferred in this study for simplicity reasons. The linear system considers each measure of equal weight, whether it poses higher or lower risk to the farm. The choice was also guided by the desire to make the scoring system comprehensive for the farmers and stakeholders of pig industry.

This study revealed that measures belonging to the three components of biosecurity were equally implemented, since the mean scores of the investigated components were not significantly different. However, within each component, some measures recorded higher scores while others were not implemented at all. For isolation component, measures with the highest score (>80) included “employees do not rear pigs at home” and “animals of different age not in the same room”. An explanation for these high scores could be that most farms have family members as workers, and in order to reduce competition for food, farmers are aware of the need to keep pigs of the same age together in the same room, rather than mixing large and small animals in the same area. The measure with the lowest score was “use of herd specific clean coveralls and boots on farm”; the lowest score is in part due to the fact that most workers do not possess dedicated clothing for farms. Effective use of coveralls and boots was observed only in intensive farms which are less represented in the study area. Thus, the low score is also likely related to the low number of intensive farms. Differences in management and biosecurity practices between smallholder and commercial (intensive) pig farms have been described also in the Philippines [24].

For measures under traffic control component, “unsold animals from market quarantined prior to reintroduction into the herd” and “production materials not exchanged among farms” were the measures with the highest score (>80) while the measure with the smallest score was “entry restriction sign post present”. Quarantine of animals from markets is a very useful biosecurity measure given that markets are public places where contact among animals from various origins and with different health status is optimal. In line with the high score concerning quarantine, it is advisable for farmers to avoid contact between own animals and other animals at the market and to disinfect any vehicle coming from the market, used to carry animals before it enters the farm. Similarly, it is very helpful not to exchange production materials (drinkers, feeders, buckets, and other tools) between farms. The high quarantine score in this study is in accordance with recommendations for biosecurity considerations on pig farms [25]. The high score observed is probably related to the awareness of farmers of the dangers associated with overlooking this measure, following the ASF outbreak and the subsequent warning and sensitization through the media (radio, televisions, and newspapers) about the main ASF transmission routes. The adoption of this measure should be sustained and encouraged not only because of ASF but also because of the zoonotic pandemic H1N1 virus (pdm/09), the etiologic agent of influenza A reported in pigs in Cameroon [26]. The fact that none of the farmers used a physical indication such as a sign post to deter people from entering the farm or from allowing pet animals to enter the farm is due to the lack of awareness of the importance of this measure. Such a practice is contrary to practices adopted by many European pig farmers whose most important biosecurity measures are those that aim at minimizing the risk of disease introduction by visits and vehicles [12, 27]. Visitors with their vehicles may unknowingly enter the farm with their pet animal such as dog or cat and reach the animal living areas where they as well as the pet animal may transmit a disease to a flock either by direct contact or by shedding the pathogen through the excreta (feces, urine, or saliva).

The measures evaluated under the sanitation biosecurity component with high score (>80) included “piggeries clean every day”, “disinfectants are used”, “pigs are vaccinated”, and “vaccination calendar respected” while that recording the lowest score was “sanitary lock is present”. Vaccination is done twice a year against erysipelas which has become endemic in the region; the vaccine used is given, according to the manufacturer's instructions, after a six-month period to growers and adults. The high score of these hygienic measures might be justified by the fact that these are basic measures easy to implement at very cheap cost. It is important to mention that, in Cameroon, vaccination of pigs and large ruminants against many contagious diseases is subsidized by the government. Also, the fear for outbreak of contagious disease such as ASF and erysipelas has pushed farmers to be receptive to advice and recommendations from the veterinary health officers and to adopt those that are cheap and easy to apply. Indeed, Fraser et al. [28] found that there is a converse relationship between the willingness of farmers to adopt a biosecurity measure and its implementing cost. However, results from recent studies showed that implementation of biosecurity measures leads to more financial profit, reduced usage of antimicrobials, and improved technical performance in pig production [29–31]. No farm was found to possess a sanitation lock in this study, though the sanitation lock is essential for keeping visitors and workers clean on the farm [25]. The null score probably has to do with financial considerations but lack of sanitary lock is contrary to recommendations for good biosecurity practices on pig farms [25].

Considering all the biosecurity measures, the score of more than half of them is below the average score of a given measure (49 points), indicating that the biosecurity level of the investigated farms is low. It is therefore important for farmers to seriously reconsider the biosecurity practices on their farms by implementing the required biosecurity measures on their farms. Previous studies evaluating the impact of the implementation of biosecurity measures in pig herds in Europe only showed advantageous results, notably decreased usage of antimicrobials, a drop in disease outbreaks, an increased technical performance, and more profit [29–31]. Policymakers should encourage farmers to implement biosecurity measures and even organize training and sensitization workshops on biosecurity in pig production.

The farm biosecurity score was significantly related to the production system, with the score lower in extensive and semi-intensive system compared with the intensive system. Our results are in tandem with previous observations [15, 32]. Some measures (avoiding introduction of pigs from outside, neighboring farm, markets or villages in the herd, ensuring long distances between farms, or shower with change of clothing and footwear, among others) implemented in the intensive system are not easily applicable in the extensive system. Thus, the relationship between biosecurity score and production system was expected given that implementation of standard biosecurity measures is limited in extensive and semi-intensive systems. Herd size is often correlated with the biosecurity score [9, 13], but in this study, this was not the case likely due to the relatively small herd sizes in general.

## 5. Conclusions

Pig farming in the western highlands of Cameroon is not the main farmers' activity, probably resulting from regular outbreaks of contagious diseases. Most farms are extensive with relatively small herd sizes and labor coming from family members. Biosecurity measures with high scores are basic (hygienic measures) or those cheap to implement. In general, the biosecurity level of the investigated farms was poor with more than half of the measures recording the score below the average score for a given measure. A significant relationship was found between farm biosecurity score and farm production system. With the results obtained in this study, stakeholders of pig industry in Cameroon and other countries with similar pig farming system can improve the general biosecurity status in pig production, which in turn will probably help in limiting the outbreak of diseases, improving the technical performance and economic gain. Policymakers should encourage farmers to implement biosecurity measures and even organize training and sensitization workshops on biosecurity in pig production in the country.

## Figures and Tables

**Table 1:** General characteristics of pig farmers and farms in Menoua Division.

Characteristics	Subdivisions			Total (*N* = 97)
	Dschang (*n* = 41)	Fokoué (*n* = 32)	Penka Michel (*n* = 24)	
Sex of farmer				
Male	24,36	78,10	75,00	76,29
Female	75,61	21,88	25,00	23,71
Farmer age *(mean ± SD)*	47.04 ± 10.18	49.34 ± 10.31	47.12 ± 10.85	47.82 ± 10.34
Education level				
Never been to school	12.20	12.50	25.00	15.46
Primary	17.07	12.50	4.17	12.37
Secondary	53.66	56.25	50.00	53.61
Higher education	17.07	18.75	20.89	18.56
Training in animal husbandry				
Yes	24.39	28.12	25.00	29.89
No	75.61	71.87	75.00	70.10
Main activity				
Crop farming	34.15	50.00	33.33	39.18
Animal husbandry	29.27	15.63	29.17	24.74
Trade	21.95	15.63	20.83	19.59
Civil servant	9.76	9.38	8.33	9.28
Retired	4.88	9.38	8.33	7.22
Religion				
Christian	82.05	80.42	75.89	79.46
Muslim	0.00	0.00	2.58	0.86
Animist	17.95	19.58	21.53	19.68
Matrimonial status				
Married	63.41	65.63	45.83	65.96
Bachelor	29.26	25.00	37.51	23.28
Widow(er)	7.32	9.37	16.66	7.76
Experience in animal husbandry (years) *(mean ± SD)*	10.75 ± 6.16	11.84 ± 6.81	9.75 ± 6.39	10.86 ± 6.42
Husbandry system^†^				
Extensive	69.25	72.64	77.78	73.22
Semi-intensive	24.89	22.55	19.89	22.45
Intensive	5.86	4.80	2.33	4.33
Herd size *(mean ± SD)*	17.14 ± 11.62	21 ± 9.89	15.21 ± 10.67	16.87 ± 11.04
Farm age *(mean ± SD)*	8.15 ± 5.39	8.84 ± 6.07	7.37 ± 5.25	8.18 ± 5.56

Apart from characteristics expressed in terms of *mean ± SD*, other characteristics are in percentage. SD = standard deviation; *N* = total number of farms. *n* = number of farms per subdivision; ^†^extensive system = animals of relatively small number are permanently penned and feed on agriculture by-products and kitchen wastes; semi-intensive system = cross-bred animals are permanently penned in piggery with a roughcast floor and feed on kitchen waste, agricultural by-products and often industrial feed; intensive system = animals are improved breeds, indoors, in high number; the piggery is a modern building; feedstuff is exclusively industrial; management system is modern.

**Table 2:** Biosecurity item score and overall score for each biosecurity component (isolation, traffic control and sanitation).

Biosecurity components					
Isolation		Traffic control		Sanitation	
Biosecurity measure	Score	Biosecurity measure	Score	Biosecurity measure	Score
Farms are fenced	41	Piggeries built based on the linear flow principle	38	Footbath is functional and used	29
Distance between two farms ≥ 500 m	58	Entry restriction sign post present	0	Piggeries broom-clean every day	90
There is only one piglets supplier for the farm	40	Each employee is assigned to a single building	74	Disinfectants are used	90
New animals are quarantined	69	Visitors not allowed to handle pigs without washing their hands	21	Sanitary lock is present	0
Animals of different age not in the same room	96	Unsold animals from market quarantined prior to reintroduction into the herd	81	Vehicles entering on farm are disinfected	4
Use of herd specific clean coveralls and boots on farm	6	Production materials not exchanged among farms	93	Shoes cleaning-station is present	25
Other animal species (fowl, sheep, goat) absent on the farm	31	Farmers do not use boars from other farms	66	Cleanout implemented	60
All-in all-out system implemented (all age categories)	23			Feedstuffs sheltered against rodents	45
Employees do not rear pigs at home	84			Drinking water is treated with chemicals (chlorine)	27
Area around farm is bush- and tree- free	16			Water and slurry drained away from the piggery	69
				Pigs are vaccinated (erysipelas)	89
				Diseases on farms are reported	42
				Vaccination calendar respected	82
				Sick animals are unsold	56
*Overall score* **(mean ± SD)**	**46.40 ± 29.7**	*Overall score* **(mean ± SD)**	**53.29 ± 34.3**	*Overall score* **(mean ± SD)**	**50.57 ± 31.1**

**Table 3:** Regression result of socioeconomic characteristics of pig farmers and technical characteristic of farms influencing the biosecurity score of farms.

Characteristics	Regression coefficient	*p*-value
*Socioeconomic characteristics of farmers*		
Age (years)	−0.004	0.953
*Gender*		
Male	0.552	0.658
Female	Ref.	
*Education*		
Higher education	1.489	0.309
Secondary education	−0.224	0.858
Primary education	0.847	0.571
Never been to school	Ref.	
*Training in animal husbandry*		
Yes answer	0.387	0.985
No answer	Ref.	
*Main activity*		
Crop producer or breeder	−3.040	0.103
Trader or civil servant	−2.108	0.315
Retired	Ref.	
*Member of a cooperative*		
Yes	0.611	0.591
No	Ref.	
*Technical characteristic of farms*		
Farm age (years)	0.121	0.270
Herd size	0.017	0.596
*Husbandry system*^†^		
Extensive system	−6.569^*∗*^	**0.000**
Semi-extensive	−3.658^*∗*^	**0.005**
Intensive	Ref.	

^*∗*^ *p* < 0.05; ^†^extensive system = animals of relatively small number are permanently penned and feed on agriculture by-products and kitchen wastes; semi-intensive system = cross-bred animals are permanently penned in piggeries with a roughcast floor and feed on kitchen waste, agricultural by-products and often industrial feed; intensive system = animals are improved breeds, indoors, in high number; the piggery is a modern building; feedstuff is exclusively industrial; management system is modern.

## Data Availability

The data are included in the tables within the manuscript.
